# Posterior Reversible Encephalopathy Syndrome (PRES) in an Anuric Patient With a WT1 Mutation and Chronic Renal Graft Failure: A Case Report

**DOI:** 10.7759/cureus.114169

**Published:** 2026-08-08

**Authors:** Charles Massinon, Willem Boer, Patrick M Honoré

**Affiliations:** 1 Intensive Care Unit, Centre Hospitalier Universitaire (CHU) UCLouvain (UCL) Dinant Godinne Namur, Chimay, BEL; 2 Intensive Care Unit, Ziekenhuis Oost-Limburg, Genk, BEL; 3 Intensive Care Unit, Centre Hospitalier Universitaire (CHU) UCLouvain (UCL) Godinne, Godinne, BEL

**Keywords:** chronic graft rejection, home hemodialysis, intensive care, pres syndrome, wt1 mutation

## Abstract

Posterior reversible encephalopathy syndrome (PRES) is a clinical and radiological entity frequently observed in solid organ transplant recipients, where it is often attributed to the neurotoxicity of calcineurin inhibitors (CNIs). However, its occurrence in anuric patients who had bilateral nephrectomy and chronic kidney disease of the graft, independent of any immunosuppressive treatment, is very rare. This case highlights the crucial and isolated role of extreme fluid overload and hemodynamic instability in triggering PRES.

A 23-year-old woman, carrying a known mutation of the Wilms tumor 1 (WT1) gene and having undergone bilateral nephrectomy in childhood followed by kidney transplantation, presented to the emergency department with altered mental status, generalized seizures, and severe hypertension. She was on intensive home hemodialysis due to chronic kidney disease of the graft. Brain magnetic resonance imaging (MRI) revealed bilateral and symmetrical T2/fluid-attenuated inversion recovery (FLAIR) hyperintensities in the insular regions, temporal lobes, and cerebellar tonsils, consistent with vasogenic edema. After ruling out infectious, ischemic, and autoimmune etiologies, a diagnosis of PRES was made. Intensive blood pressure control and optimized fluid management resulted in rapid neurological recovery and near-complete resolution of the radiological lesions.

In this case, significant volumetric and hemodynamic fluctuations alone can disrupt cerebral autoregulation and trigger PRES in anuric patients, even years after immunosuppressant discontinuation. Clinicians must remain vigilant for the possibility of PRES in dialysis patients with acute neurological symptoms, as rapid management of blood pressure and fluid balance is essential to ensure reversibility and prevent permanent neurological sequelae.

## Introduction

Posterior reversible encephalopathy syndrome (PRES) is a clinical-radiological entity characterized by acute neurological symptoms, including severe headaches, seizures, visual disturbances, and altered mental status. Recent data show that chronic kidney failure is a primary driver for PRES in 50% of cases, although the exact pathophysiology remains debated [1].

Among young adults with kidney transplants, the syndrome is often attributed to the neurotoxicity of CNIs [2]. But in the context of Wilms tumor 1 (WT1), early-onset renal failure often requires bilateral nephrectomy in childhood. In this condition, patients become totally dependent on extra-renal purification for fluid and pressure homeostasis until they receive a kidney transplant. 

Following chronic graft rejection from a previous transplant, patients returning to intensive dialysis face some significant hemodynamic challenges. We report a rare case of PRES in a 23-year-old female with chronic rejection of the renal graft, illustrating that volumetric instability can trigger PRES independently of immunosuppressive drugs.

## Case presentation

A 23-year-old female was admitted to the emergency department because she lost consciousness and vomited at home. She has also had recurrent headaches for approximately two months. Her relatives have reported incoherent speech, memory loss, and confusion for 12 hours before her admission. In her medical history, she was diagnosed with congenital nephrotic syndrome associated with a confirmed WT1 gene mutation.

A mutation in the WT1 gene disrupts a key tumor suppressor gene located on chromosome 11, which normally regulates kidney and reproductive development. These alterations disrupt the control of cell growth, leading to congenital disorders or increasing the risk of kidney cancer in children (Wilms tumor).

She underwent a bilateral nephrectomy followed by a first renal transplantation. After a decade of graft function, she developed chronic graft rejection, which necessitated a return to renal replacement therapy by home hemodialysis (HDD), performed five times per week. Due to her kidney failure, she had hypertension that is difficult to control with several antihypertensives ( clonidine, atenolol, perindopril, and terazosin).

Upon her admission to the emergency room, blood pressure was 250/190 mmHg, heart rate 115/min, respiratory rate 28/minute, oxygen saturation 96% on room air, Glasgow coma scale (GCS) 9/15 (eye response spontaneous, no verbal response, withdrawal from pain for motor response), and random blood sugar 122 mg/dl (70.0-100.0 mg/dl).

Her examination showed clonic movements of the upper limbs, reaction only to pain, stuporous states, open eyes and bilateral isoreactive mydriatic pupils, and no neck stiffness. Cardiac, respiratory, and abdominal examinations were unremarkable. Blood gas analysis was performed on admission, and thorough hematological and biochemical investigations were done. 

Arterial blood gases showed mild hypercapnea, hyperlactatemia, and hyperkalemia with a normal pH (Table 1).

**Table 1:** Blood gases on admission pCO2: partial pressure of arterial carbon dioxide; pO2: partial pressure of arterial oxygen; HCO3: arterial bicarbonate concentration; BE: base excess

Parameters	Patient admission value	Reference range
pH	7.36	7.35-7.45
pCO2 ( mmHg)	52	35-45
pO2 (mmHg)	83	75-100
HCO3- (mEq/L)	27.0	22-28
BE (mM/L)	2.8	-2 to 3
Potassium (mM/L)	5.8	3.5-4.5
Lactate (mM/L)	5.6	0.5-2.0

Laboratory tests revealed mild polycythemia, no signs of inflammation, severe renal impairment (creatinine level was 12.38 mg/dl) with hypernatremia and mild hyperkalemia, severe hyperphosphatemia, no liver dysfunction, negative toxicological screening, and a negative pregnancy test (Table 2).

**Table 2:** Laboratory tests at admission eGFR: estimated glomerular filtration rate; MDRD: Modification of Diet in Renal Disease study; GOT: glutamic oxaloacetic transaminase; GPT: glutamate pyruvate transaminase; LDH: lactate dehydrogenase; CPK: creatine phosphokinase; ALP: alkaline phosphatase; GGT: gamma-glutamyl transferase

Parameters	Patient admission value	Reference Range
Hemoglobin (g/dl)	15.8	12.9-16.7
White blood cells ( x10³/µL)	14.24	3.8-9.8
Neutrophils absolute (x10³/µL)	7.13	1.4-5.9
C-reactive protein ( mg/L)	2.4	0.0-5.0
Urea (mg/dl)	97.8	18-55
Creatinine (mg/dl)	12.38	0.72-1.25
eGFR (MDRD)	4.0	60.1-200.0
Phosphate (mmol/L)	3.20	0.81-1.45
Sodium (mmol/L)	148	136.0-145.0
Chloride (mmol/L)	95	98.0-107.0
Potassium (mmol/L)	5.52	3.5-5.1
Total calcium (mmol/L)	2.93	2.2-2.6
Bicarbonate (mmol/L)	16.4	23.0-31.0
Magnesium (mmol/L)	1.60	0.66-1.07
GOT (U/L)	24	5.0-34.0
GPT (U/L)	10	0.0-55.0
LDH (UI/L)	372	125.0-220.0
CPK (UI/L)	50	30.0-200.0
ALP (UI/L)	62	44.0-107.0
GGT (UI/L)	22	0.0-38.0
Total serum bilirubin (mg/dl)	0.25	0.2-1.2

**Figure 1:**
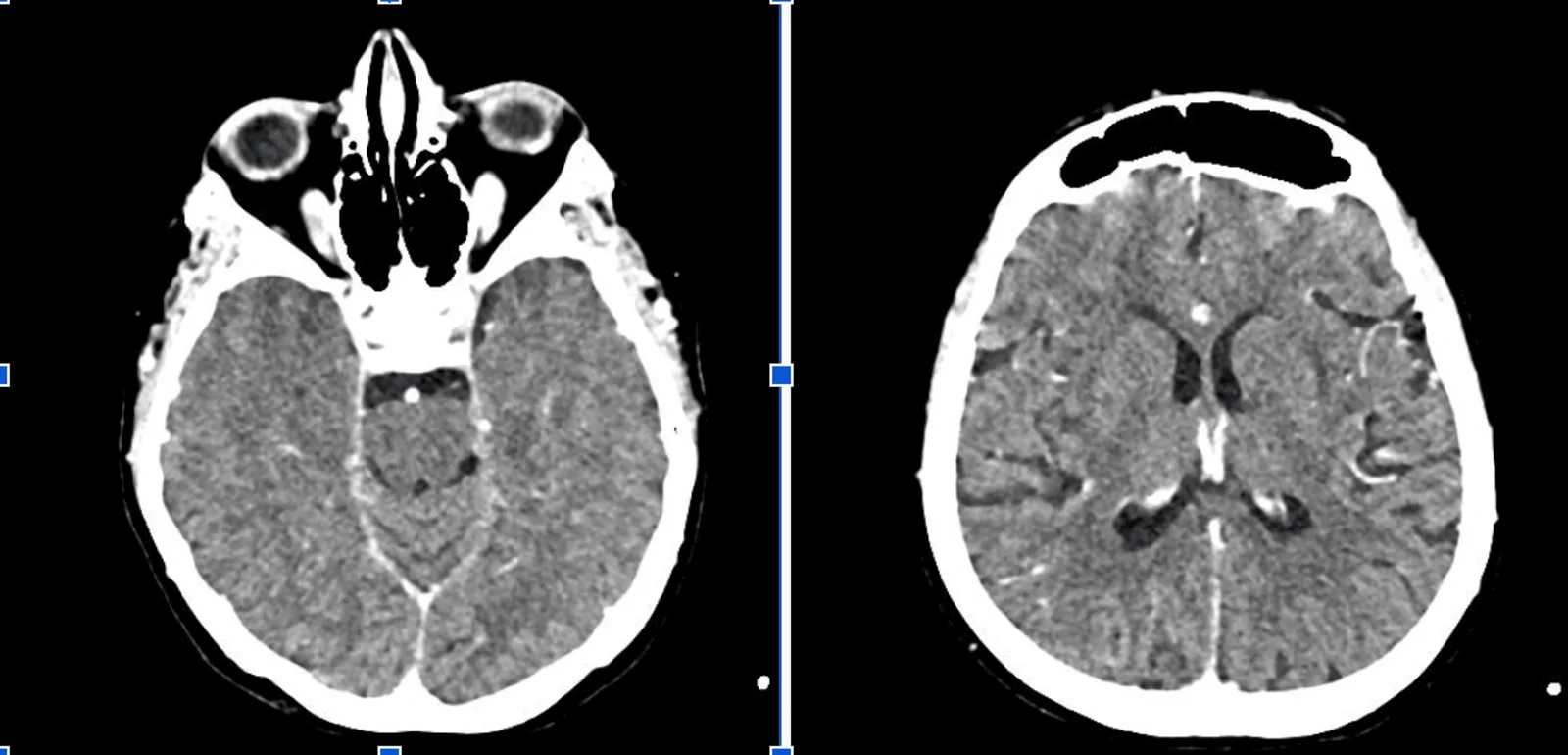
Cerebral CT angiography showing no arterial abnormality and no pathological parenchymal enhancement in the supratentorial region

Emergency cerebral angiography was performed to rule out a cerebral hemorrhage and an arterial or venous occlusion, but was unremarkable (Figure 1).

Due to her neurological condition and to protect her airway, the patient was intubated and admitted to the intensive care unit (ICU) for neurologic and hemodynamic monitoring. Upon her admission to the ICU, a cerebrospinal fluid (CSF) exam was performed, which showed 0 nucleated cells, 10 red blood cells, and mild hyperproteinorachia. Glucose and lactate were within normal ranges; multiplex PCR analysis excluded infectious etiologies (Table 3).

**Table 3:** Cerebrospinal fluid analysis at admission

Parameter	Result	Reference Range
Nucleated cells ( µ/L)	0	10-20
Glucose (mg/dl)	60.7	40.0-70.0
Protein (mg/dl)	51.5	15.0-40.0
Lactate (mg/dl)	18.5	10.0-18.9
Multiplex polymerase chain reaction panel	Negative	Negative

An electroencephalogram (EEG) was performed and showed severe encephalopathy with slow-wave activity overload bilaterally in the temporal lobe without epileptic activity. Even though no epileptic activity was detected on the EEG, anti-epileptic treatment with levetiracetam was initiated. The patient was sedated with propofol, and her blood pressure was controlled by continuous intravenous infusion of nicardipine and clonidine, allowing for a gradual reduction in his blood pressure.

**Figure 2:**
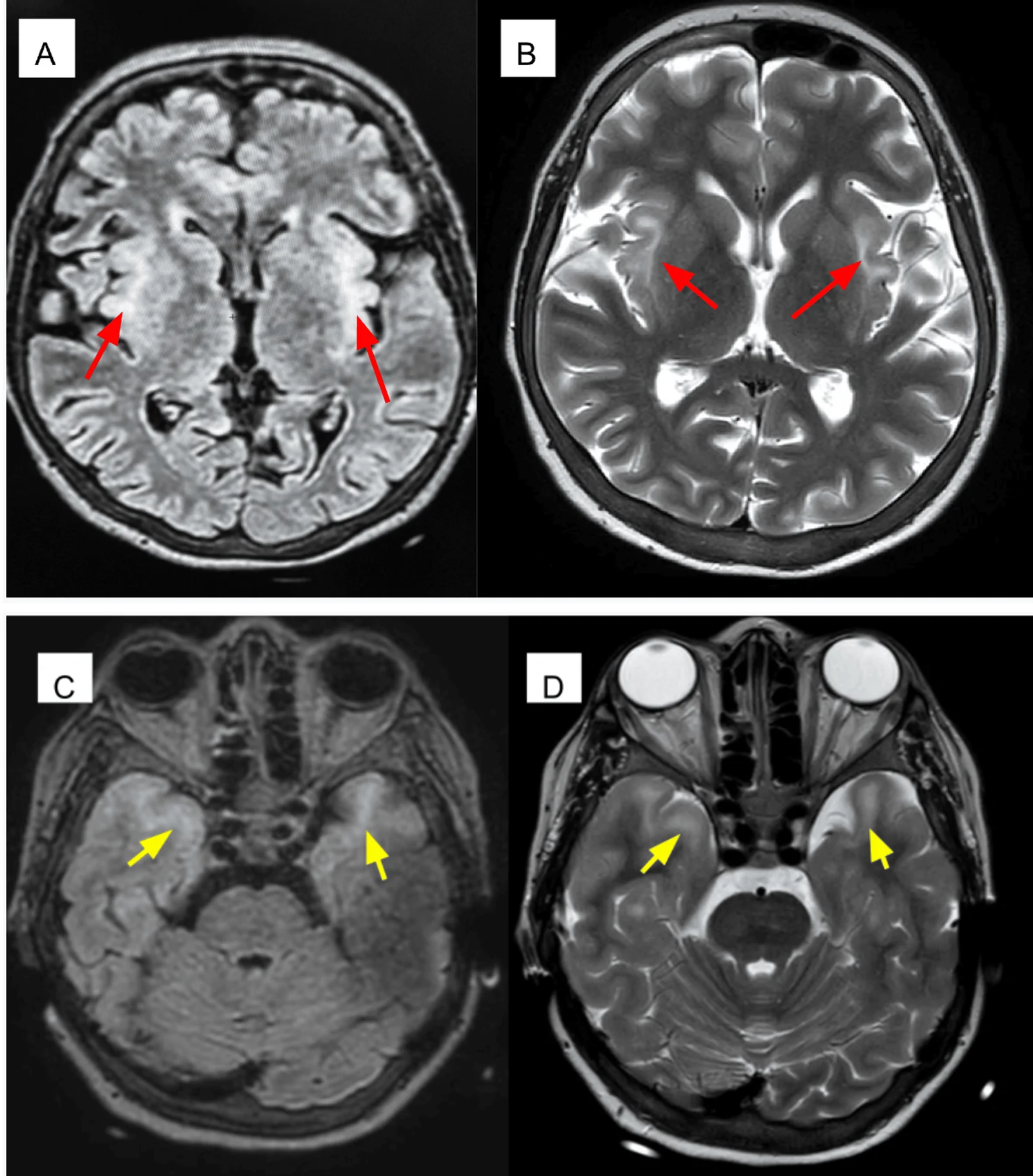
Initial brain magnetic resonance imaging (MRI) of a 23-year-old female patient Axial fluid-attenuated inversion recovery (FLAIR) (A, C) and T2-weighted (B, D) images of the hemisphere. There were hyperintense lesions in the insular and temporal areas consistent with vasogenic edema (red arrows). There were also hyperintensities in both amygdalae (yellow arrows).

On day 2 of admission, a brain magnetic resonance (MRI) was performed and demonstrated bilateral and symmetrical T2/fluid-attenuated inversion recovery (FLAIR) pathological hyperintensity of the insular areas and temporal lobes, particularly the hippocampi and amygdala, without enhancement on gadolinium injection. There was no lesion hyperintensity on diffusion-weighted imaging, and no signs of bleeding were detected (Figure 2).

A differential diagnosis of acute neurological involvement suggested several possibilities: autoimmune disease, herpes encephalitis, metabolic encephalitis, ischemic origin, dialysis disequilibrium syndrome (DDS), and PRES syndrome. Because of the MRI findings, the radiologist initially suspected herpetic encephalitis. The neurologist agreed, and antiviral therapy with acyclovir was initiated.

A second CSF analysis was performed 24 hours after the first, with analyses for autoimmune abnormalities. The autoimmune encephalitis panel, onco-neuronal antibodies, and oligoclonal band were negative. PCR for viral etiologies was negative too.

Antineutrophil cytoplasmic antibodies (ANCA) and anti-glomerular basement membrane (GBM) antibodies were negative, making vasculitis less likely. Aciclovir was stopped after two negative CSF tests. Given the possible autoimmune origin and pending the results of the autoimmune panel tests, a pulse corticotherapy was started on day 3 with a dose of 1g per day for 5 days.

On day 3, she was weaned off the ventilator. Intravenous antihypertensives were slowly reduced, and intravenous medications were switched to oral administration. On day 4, her neurological status improved rapidly, but mood disturbances with frequent crying remained present, as well as memory loss.

**Figure 3:**
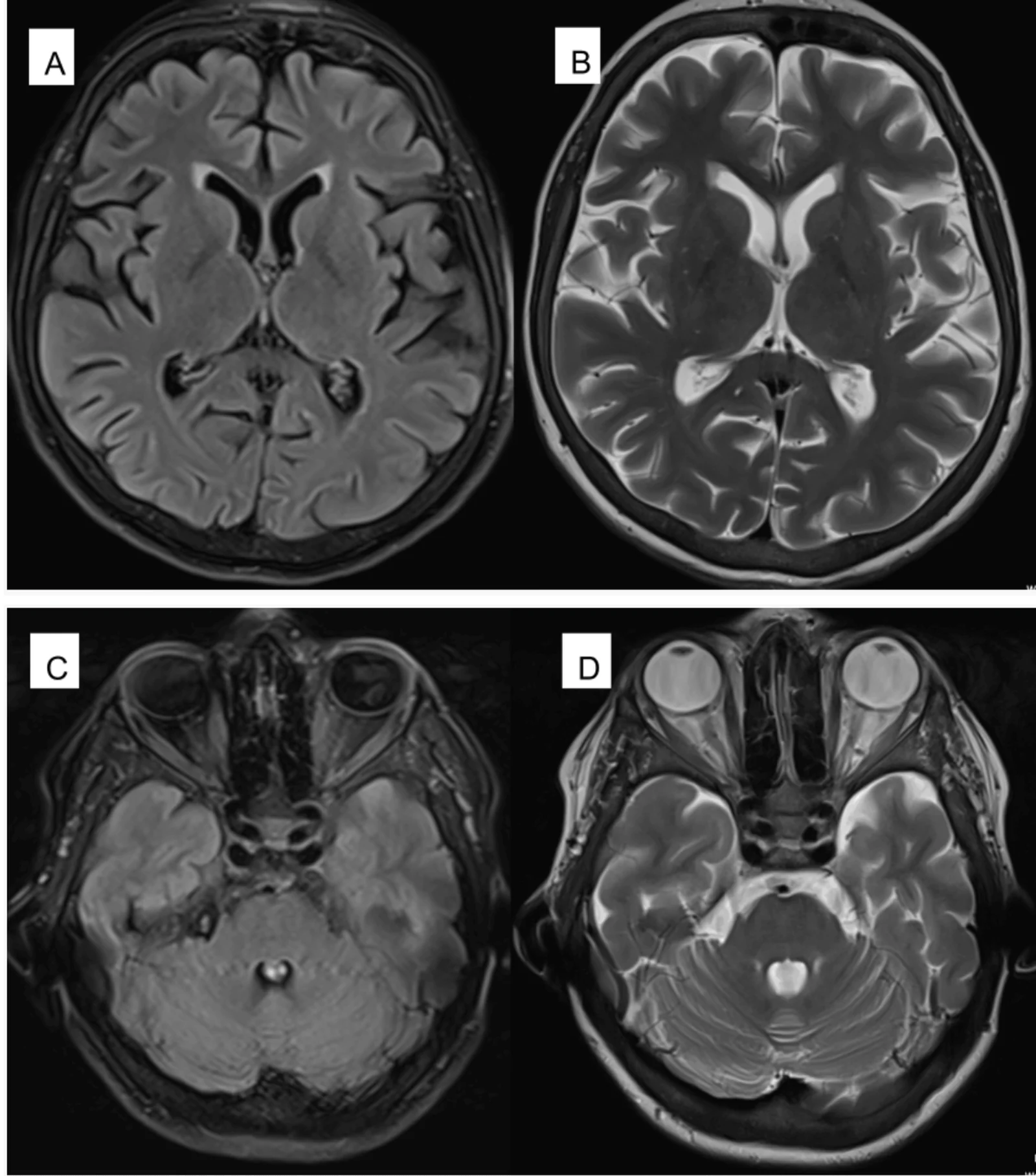
Brain MRI on day 60 after onset Axial fluid-attenuated inversion recovery (FLAIR) and axial T2-weighted hemisphere (A, B) images, as well as axial FLAIR and T2-weighted posterior fossa images (C, D), show near-resolution of the lesions.

The patient continued to improve over the next few days; she was discharged from the ICU on day 7 and transferred to the neurology department. On day 10, she was able to leave the hospital and return home. A follow-up MRI brain showed resolution of the lesions on day 60 after onset (Figure 3).

Six months later, she was the beneficiary of a second kidney transplant, which permitted her to recover normal kidney function and regain satisfactory diuresis, allowing her to stop hemodialysis sessions and reduce the intensity of her antihypertensive treatment. 

## Discussion

PRES is an acute neuro-radiological entity characterized by headache, visual disturbances, seizures, and altered mental status, with symptoms evolving over hours to days [3-5].

Many risk factors can lead to PRES, including acute hypertension, preeclampsia/eclampsia, anti-neutrophil cytoplasmic autoantibody‑associated vasculitis, systemic lupus erythematosus, solid organ transplantation, chemotherapy or cytotoxic drugs, and renal insufficiency.

Chronic kidney disease (CKD) is a known but not exclusive cause of its development, but the relationship between CKD and PRES remains poorly understood and is currently debated [1,6]. In a retrospective observational study, Canney et al. reported an incidence of 0.84% in this population [7]. The specificity of this case is linked to a rare and complex medical history of a young female with a congenital nephrotic syndrome caused by a WT1 gene mutation, who developed PRES in the context of home hemodialysis while awaiting a new kidney transplant. Kidney transplant recipients have many risk factors that can lead to PRES; one of these triggers is the use of calcineurin inhibitors (CNIs), especially tacrolimus [2]. But the patient was not receiving CNIs and was maintained on a minimal dose of prednisolone (3 mg/day); this dosage is essentially physiological to avoid adrenal insufficiency and to control chronic inflammation linked to the non-functional graft.

The pathophysiology of PRES remains debated, but the central element is dysfunction of the blood-brain barrier (BBB), which maintains homeostasis [3,5,8,9]. When this barrier is compromised, there is a leakage of plasma components into the interstitial space, which produces cerebral vasogenic edema. This dysfunction is primarily linked to two mechanisms: endothelial injury and impaired autoregulation of cerebral blood flow, which is linked to severe hypertension [8,9].

In patients with the WT1 gene mutation, there is often an association with Denys-Drash and Frasier syndromes, but this association is not limited exclusively to the renal and oncological spheres. Beyond the kidney, the WT1 gene plays a crucial, though less-discussed, role in vascular health [10]. It is generally accepted that WT1 is a critical regulator of the renin-angiotensin system; its mutation promotes severe, early-onset systemic hypertension. But what is less known is the crucial role of WT1 in directly regulating the transcription of vascular endothelial growth factor (VEGF) [11].

In healthy states, WT1 maintains adequate VEGF levels necessary for endothelial cell survival and vessel integrity, including the BBB. A loss-of-function mutation in WT1 may lead to "VEGF-depletion" like states, mimicking the effects of anti-angiogenic drugs (e.g., Bevacizumab), which are known iatrogenic causes of PRES [12].

In animal models, WT1 deficiency has been linked to impaired barrier functions in the central nervous system, specifically within the choroid plexus [13]. All of these elements suggest that WT1 mutations induce intrinsic endothelial fragility. The resulting dysregulation of the neurovascular unit diminishes the brain's homeostatic capacity to withstand fluctuating perfusion pressures, thereby lowering the threshold for vasogenic edema.

A key point in our patient's clinical situation is the progressive loss of renal graft function, necessitating a return to dialysis. She was placed on a short daily home hemodialysis (HHD) program to optimize volume status and metabolic control, performed five times per week. Daily dialysis with smaller volume variations is certainly more likely to be beneficial than dialysis three times a week with larger variations, but high-frequency hemodialysis can also, counterintuitively, create a state of permanent hemodynamic instability by exposing the patient to rapid changes in plasma osmolality and recurrent fluid fluctuations [1,14]. Furthermore, chronic graft rejection creates a chronic inflammatory state in the blood vessels.

In patients with a WT1 genetic background, who present a long-term weakened endothelium, the cerebral autoregulatory window is exceptionally narrow [10,11]. The combination of genetic profile, inflammatory state related to transplant rejection, and hemodialysis leads to an increased risk of dysfunction in cerebral perfusion autoregulation.

In this case, the definitive trigger was the malignant hypertension with a measured blood pressure of 250/190 mmHg. At this extreme pressure level, the cerebral autoregulation system entirely collapsed. The protective reflex vasoconstriction of the cerebral arterioles failed under mechanical stress, leading to forced hyperperfusion and massive vasogenic edema [4,9,15].

The management of PRES relies primarily on correcting precipitating factors, with particular attention to blood pressure control. Patients with malignant hypertension should benefit from a gradual reduction with a target mean arterial pressure between 125 and 105 mmHg [3,5,15]. Other key points include seizure control, ideally with continuous EEG monitoring.

Treatment of infections (typically herpes simplex virus infections)and reduction or discontinuation of potentially implicated medications are important. Renal replacement therapy should be initiated if indicated and delivery should take place where feasible in obstetric cases.

PRES remains a diagnostic challenge due to the wide variety of clinical presentations and the diversity of radiological abnormalities. Many acute neurological conditions can resemble PRES; these include stroke, epilepsy, infectious encephalitis, vasculitis, toxic or metabolic encephalopathies and rarer entities [1,3].

Neuroimaging is an essential element in the diagnosis of PRES, particularly MRI, which allows the detection of vasogenic edema, especially on T2-weighted and FLAIR sequences, which appear as typical hyperintense lesions [3,15].

In our clinical case, once the patient returned home, managing her hypertension remained difficult despite a combination of different medications, the successful completion of the hemodialysis sessions, and the patient's therapeutic compliance. It was only after her second kidney transplant that the patient regained good hemodynamic control.

## Conclusions

This clinical case highlights that posterior reversible encephalopathy syndrome (PRES) can manifest as a catastrophic complication of a malignant hypertensive crisis in young patients with a WT1 genetic background. A phenomenal blood pressure spike of 250/190 mmHg constitutes a distinct and definitive mechanism of complete failure of cerebral autoregulation and the resulting massive vasogenic edema. Furthermore, this clinical presentation shows a critical paradox: an intensified, high-frequency home hemodialysis protocol may not fully protect highly vulnerable patients from severe hemodynamic fluctuations during the critical period of progressive renal graft failure.

This report provides an essential lesson: the WT1 mutation, combined with the systemic stress associated with graft failure, creates an extremely narrow cerebral vascular tolerance margin. In the event of neurological symptoms in this specific population, PRES should be immediately suspected, regardless of immunosuppressive therapy or dialysis frequency. Early radiological recognition by MRI, followed by aggressive but controlled blood pressure reduction and meticulous optimization of fluid status, remains essential to achieve complete clinical reversibility and prevent permanent neurological sequelae or fatal outcomes.
